# Effect of Deformation Temperature on the Mechanical Behavior and Stability of Retained Austenite in TRIP-Assisted Medium-C Multiphase Steel

**DOI:** 10.3390/ma13112433

**Published:** 2020-05-26

**Authors:** Adam Skowronek, Adam Grajcar

**Affiliations:** Department of Engineering Materials and Biomaterials, Silesian University of Technology, 18A Konarskiego Street, 44-100 Gliwice, Poland; adam.skowronek@polsl.pl

**Keywords:** TRIP steel, medium-C steel, retained austenite, deformation temperature, mechanical stability

## Abstract

The temperature-dependent microstructural evolution and corresponding mechanical stability of retained austenite in medium-C TRIP-assisted 0.43C-1.45Mn-0.98Si-1Al-0.033Nb-0.01Ti steel obtained by thermomechanical processing was investigated using static tensile tests and microstructural studies. The light microscopy, image analysis, XRD diffraction and the Jaoul–Crussard analysis were applied to reveal relationships between microstructure and mechanical properties. Specimens were deformed in the static tensile tests in a temperature range of −20–140 °C. It was found that an increase in deformation temperature resulted in the reduced intensity of the TRIP effect due to the higher stability of retained austenite. An increase in the retained austenite stability along with a smaller grain size and a change from its blocky morphology to thin layers was also indicated. The impact of strengthening mechanisms at different temperatures was analyzed. The best combination of strength and ductility was obtained in the samples deformed at 20 and 60 °C, which is associated with the moderate work hardening in this temperature range. The Jaoul–Crussard analysis showed much less strengthening during the second phase of deformation at 100 and 140 °C due to the high stability of retained austenite. The higher C content in the investigated TRIP steel resulted in substantial volume fractions of retained austenite stable after completing deformation.

## 1. Introduction

Safety is a priority in the modern automotive industry. From year to year, requirements regarding strength of the car structure and its ability to absorb energy released during road accidents are increasing. Advanced high strength steels (AHSS) have become the basic materials for the most responsible body parts. The first generation of AHSS, which consists of the dual phase (DP), complex phase (CP), hot-formed (HF), martensitic (MART) or transformation induced plasticity (TRIP) steels, is very well developed at the moment, which allows us to use a wide range of properties corresponding to various requirements. Among the steels of this generation, the TRIP steels are the most promising [1,2].

The strain-induced martensitic transformation of retained austenite provides mechanical properties not found in other materials used in the automotive industry. The microstructure of these steels contains 50–60% ferrite, 25–40% bainite and 5–15% retained austenite [3]. The variation in the selection of a microstructure composed of these phases allows the production of steel with a wide range of properties: yield strength (YS) from 500 to 1200 MPa and total elongation (TEl) from 15% to 40%. The main disadvantage of conventional, ferritic-based TRIP steels is their reduced susceptibility to stretch flangeability and bendability [4]. It has been described that these restrictions can be minimized by increasing the homogeneity of the microstructure and by reducing the hardness difference between structural constituents. The homogeneity can be obtained by precipitation hardening and grain refinement, which is accomplished by Ti/Nb/V microadditions and thermomechanical treatment [3,5]. Limiting large differences in hardness can be done by replacing a part of the soft ferrite matrix by increasing the proportion of the bainitic phase. It also affects the relationship of strength and plastic properties towards increasing the yield and tensile strengths. Increasing the fraction of bainite can be obtained, among others, by increasing a content of carbon [6,7]—the main element stabilizing the austenite in steel. However, an excessive increase in the carbon content deteriorates the weldability [8,9], which is unacceptable in industrial applications. Such steels with the increased C contents are also used for forgings and wires [2,10].

Both the strength and ductility of TRIP steel are mainly controlled by the amount of retained austenite. In turn, the critical characteristic of this phase is its stability. It affects the martensitic transformation susceptibility of the austenite and is controlled both chemically, mechanically and thermally. The chemical stabilization is based on the enrichment of austenite mainly in carbon (and manganese, in the case of medium-manganese steels [11,12,13]). A lot of works have been investigating the impact of subsequent heat treatment stages on a carbon redistribution and thus austenite stabilization [6,14,15,16,17]. The mechanical stability is another important factor. It is affected by the phase composition [18,19,20], grain size [15,19,21,22] and the steel morphology [23,24,25]. These factors depend on what stress state and strain rate will be required for the martensitic transformation to occur [16,21,26,27,28,29].

The thermal stability is directly related to both chemical and mechanical stabilities. It depends on whether the material will undergo the TRIP effect at a given temperature. When designing steel for body parts, it is necessary to consider the sheet’s forming and operating conditions. The retained austenite in TRIP steel must be stable enough to ensure that the martensitic transformation does not occur during the cooling of the material to room temperature. So, the martensitic start temperature (M_s_) of the steel should be several dozen degrees below zero. Steel with the TRIP effect during forming is exposed to sudden temperature increases [30] due to friction and deformation heat released during pressing operations. During exploitation, however, vehicles in European climatic conditions are also exposed to a wide range of temperatures, both positive in summer and negative in winter. Bearing these factors in mind, it is important to consider how the stability of the retained austenite changes at different periods of product life when designing steel. During pressing, the temperature parameters of the process can be set so that only part of the retained austenite undergoes transformation, and the remaining part can be used as an energy absorber during the collision. However, at temperatures below zero the material may show reduced properties, causing the TRIP effect to occur too rapidly preventing the proper energy absorption. Only a few papers on this subject have been reported so far [21,31,32,33] proving that it is not sufficiently studied. Thus, the present paper aims at determining changes in the mechanical stability of retained austenite (γ_R_) at different deformation temperatures, which reflect technological windows of manufacturing conditions (sheet stamping, bending, etc.) or service conditions at decreased or elevated temperatures.

## 2. Materials and Methods

### 2.1. Material and Processing Parameters

The chemical composition of the investigated material is presented in Table 1. The Nb and Ti microadditions were added to increase the strength through grain refinement and precipitation hardening. The increased carbon content compared to typical TRIP steels (0.15–0.25 wt %) was intended to further facilitate the enrichment of γ_R_ with this stabilizing element, while creating a certain portion of bainitic phase. The material was produced in a vacuum induction furnace and cast into ingots. Then the material was forged to obtain a thickness of 22 mm and roughly rolled in the temperature range of 1200–900 °C to obtain a sheet with a thickness of 4.5 mm.

**Figure 1:**
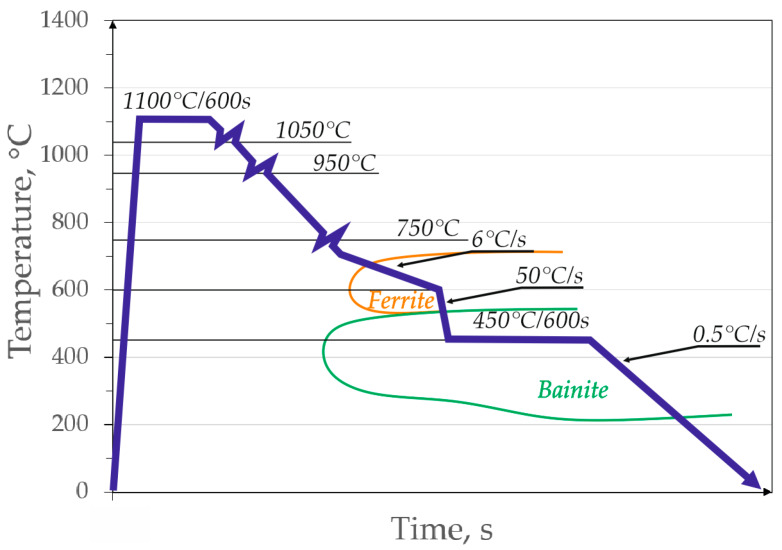
Scheme of thermomechanical processing of the investigated steel.

The last stage of production, leading to the required microstructure and properties was thermomechanical rolling (Figure 1). In the first stage, the austenitization was performed at 1100 °C for 600 s. Then the material was rolled in three passes at 1050 °C, 950 °C and 750 °C to obtain a final thickness of 2 mm. After rolling, the material was cooled at a rate of 6–600 °C/s to obtain some fraction of ferrite, and next at a rate of 50–450 °C/s. The next stage of treatment was isothermal holding within 600 s followed by cooling of the material to room temperature.

### 2.2. Static Tensile Tests

**Figure 2:**
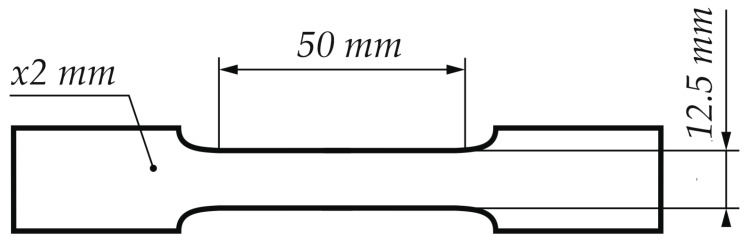
Main dimensions of the static tensile test specimens in accordance with ASTM E8/E8M.

To determine the effect of temperature on strain-induced martensitic transformation, a static tensile test using Zwick Z/100 testing machine (Zwick Roell, Ulm, Germany) was performed at a strain rate of 5 × 10^−3^ s^−1^ at temperatures in the range from −20 to 140 °C. Flat samples (Figure 2) of 50 mm gauge length, 12.5 mm width and 2 mm thick were cut from the sheet along the rolling direction. The required test temperature was ensured by using an environmental chamber. The test temperature range has been selected to best correspond to the real operating conditions of the material. In order to equalize the temperature across the entire cross-section of the material, the samples were initially held at the deformation temperature for 30 min.

The calculations were performed to determine the work hardening exponent. The true stress and true strain were calculated using the results of a static tensile test, based on the equations:

(1) $$\;\sigma =\frac{F}{S}=\sigma_{p}\left(1+\varepsilon_{p}\right),\;\mathrm{MPa}$$

(2) $$\;\varepsilon =\ln\left(1+\varepsilon_{p}\right)$$

where: *σ*—true stress, *ε*—true strain, *F*—load, *S*—true cross-section of the sample, *σ_p_*—engineering stress and *ε_p_*—engineering strain.

The determination range of the instantaneous work hardening exponent from a true stress corresponding to the yield strength up to the maximum value of tensile stress, which corresponds to the initiation of the neck formation in the sample is expressed by the equation:

(3) $$n=\frac{\varepsilon d\sigma }{\sigma d\varepsilon }=\frac{d\left(log\sigma \right)}{d\left(log\varepsilon \right)}$$

where: *n*—work hardening exponent, *σ*—true stress and *ε*—true strain.

In order to identify individual mechanisms responsible for strengthening the tested steels, a Jaoul–Crussard analysis [34,35] was performed consisting in the analysis of true stress–strain curves. This analysis is based on the following equations:

• Ludwik model [36]:

(4) $$\sigma =\sigma_{0}+K\prime \varepsilon^{n},\;\mathrm{MPa}$$

(5) $$log\left(\frac{d\sigma }{d\varepsilon }\right)=\log K\prime +\;log\;n^{\prime }+\left(n^{\prime }-1\right)\log\varepsilon ,\;\mathrm{MPa}$$

• Swift model [37]:

(6) $$\sigma =K_{s}\left(\varepsilon +\varepsilon_{0}\right),\;\mathrm{MPa}$$

(7) $$\log\left(\frac{d\sigma }{d\varepsilon }\right)=\log\left({n\prime \prime }^{{K^{\prime \prime }}^{\frac{1}{n^{\prime \prime }}}}\right)+\left(1-\frac{1}{n^{\prime \prime }}\right)log\sigma ,\;\mathrm{MPa}$$

where: *σ_0_*—initial true stress, *ε_0_*—initial true strain, *K’* and *K’’*—material constants and *n’* and *n’’*—work hardening exponents.

The equations mentioned above are described in greater detail and graphically presented by Hertelé et al. [38]. The Jaoul–Crussard analysis allows for the determination the number and impact of characteristic strengthening mechanisms in the overall strain hardening of the material.

### 2.3. Microstructural Study and Image Analysis

Initial microstructure and changes in the microstructure at various deformation temperatures were analyzed using light (Leica MEFa) microscopy (Leica Microsystems, Wetzlar, Germany). The samples for the microstructural analysis were cut from the necking area. They were prepared by using standard metallographic procedures. The 3% nital and 10% sodium pyrosulfate reagents were used to reveal ferrite grain boundaries and other microstructural constituents. This etching method due to selective contrasting allows for distinguishing individual structural constituents [39].

The final stage of microstructural observations was the image analysis of etched samples using Image-Pro Plus 6.0. (Media Cybernetics, Rockville, MD, USA). This process allowed us to determine the surface share of the individual phases and their morphology based on differences in colors obtained during etching. The analysis of austenite grains transformed into martensite processed in a 0–1 manner. Partially transformed grains were considered as the transformed ones (1). The quantity of retained austenite grains transformed into martensite was relatively compared to the retained austenite quantity in the initial microstructure (100%). The images from the light microscope have been transformed into binary maps allowing the calculation of the surface fraction and quantity of individual phase grains and better visualization of the phases. Creating a binary map first consists of converting the image to grayscale. The next stage is to narrow the scale of shades to the area that has the phases we are interested in. After indicating the shades of the phase of interest, the program further converts the image to the final form of the binary map. The image analysis is a relatively inaccurate study because the results depend on the interpretation of the colors due to the etching. However, all analyses performed under the same conditions affect good comparability of the obtained results. For each microstructure, the analysis was performed for three areas. The average was calculated from the results.

Based on the results of image analysis, a logarithmic change of the retained austenite amount in steel as a function of true strain was calculated showing the mechanical stability of this phase, expressed by the *k_s_* coefficient. For this purpose, the following equation was used [40]:

(8) $$logS_{y}=logS_{y0}+k_{s}\varepsilon$$

where: *k_s_*—constant determining the mechanical stability of the retained austenite; *S_γ_*—surface fraction of the retained austenite at strain *ε* and *S_γ0_*—initial surface fraction of the retained austenite.

### 2.4. X-ray Diffraction

In order to determine the accuracy of the results obtained during image analysis, an X-ray analysis based on the Rietveld method was performed. X-ray investigations were performed using an Empyrean Panalytical diffractometer (Malvern Panalytical, Malvern, UK) working at voltage of 40 kV and a current of 30 mA. The Co source with an iron filter in configuration with a Pixel detector was applied. The registration of diffraction signal was performed for the 2Θ angles ranging from 45 to 110°.

## 3. Results

### 3.1. Mechanical Properties

**Figure 3:**
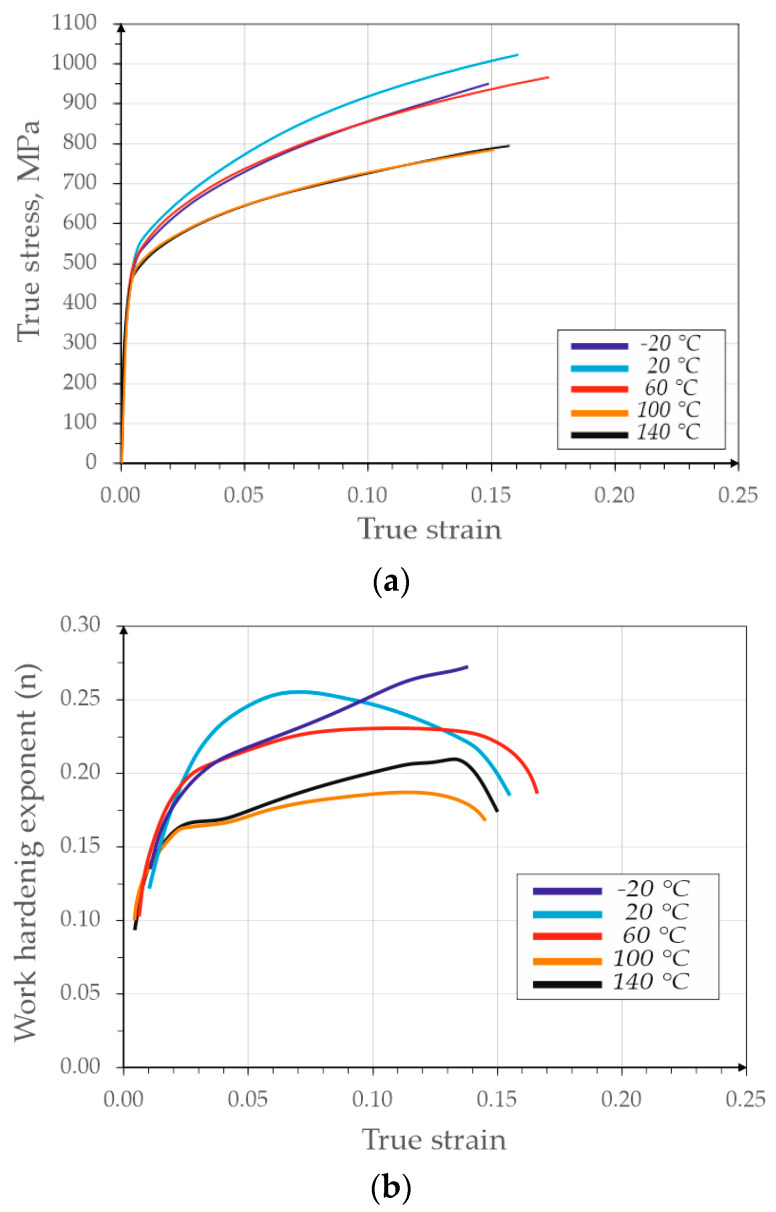
Mechanical properties of steel deformed at different temperatures: (**a**) true stress–strain curves and (**b**) work hardening exponent (*n*) as a function of true strain.

The mechanical properties for various deformation temperatures developed on the basis of the static tensile test curves (Figure 3a) are presented in Table 2. A clear influence of the temperature on the analyzed mechanical properties was visible. As the temperature increased, both YS and ultimate tensile strength (UTS) decline tendencies fell, whereas total elongation increased. The only exception is the temperature −20 °C, at which simultaneously the strength and plastic properties were smaller compared to the room temperature. The samples deformed at 20 and 60 °C show the best combination of mechanical properties. They are characterized by (UTS) at the level of 890 and 818 MPa and elongation respectively 18.3% and 19.1%. The true stress–strain curves indicate that the strengthening results in the strength at a level from about 800 MPa for the samples deformed at 100 and 140 °C to above 1000 MPa for the sample deformed at 20 °C. The temperature strongly influences the work hardening exponent (Figure 3b). The sample deformed at the lowest temperature shows a moderate increase of the *n* exponent throughout the strain range. At the initial stage, this is the desired effect increasing the material’s susceptibility to forming operations.

However, at a later stage, this results in the rapid strengthening of the material resulting in the sample fracturing during the formation of the neck. This is the reason for the lowest elongation of the deformed sample at −20 °C. The sample deformed at 20 °C is characterized by the most intense growth of *n* exponent in the first phase. However, after reaching the maximum at a true strain of 0.065, this value begins to decline. The sample deformed at 60 °C shows stabilization of *n* value after the initial growth. Moderate hardening results in the highest elongation.

**Figure 4:**
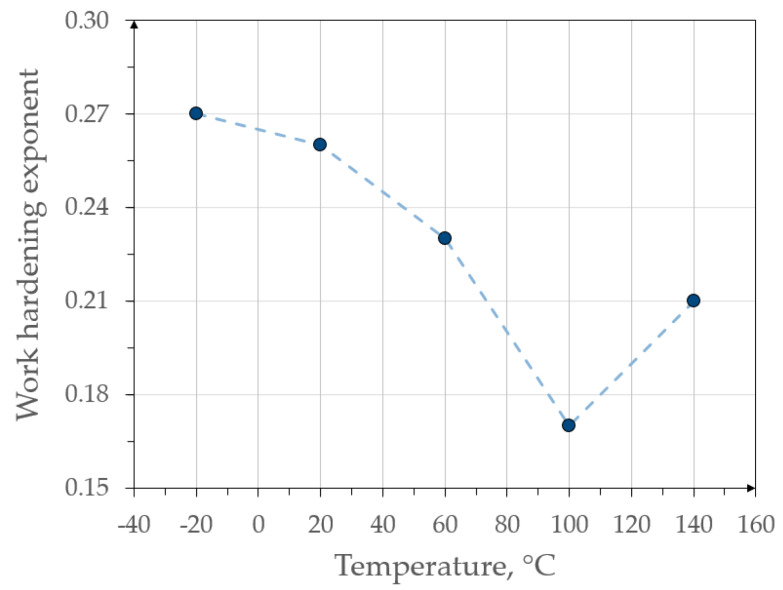
Maximum values of work hardening exponent at various temperatures.

The samples deformed at 100 and 140 °C show the *n* exponent course similar to the first sample, except that the values of *n* are much smaller. At the end of the test the strengthening begins to decrease due to the formation of the neck. They show high elongation but at the cost of a large loss of strength. There is a visible tendency of the maximum value of the work hardening exponent to decrease with the temperature variation (Figure 4). This indicates that the mechanisms of steel strengthening like the TRIP effect are limited as the temperature increases.

### 3.2. Microtructural Behavior

**Figure 5:**
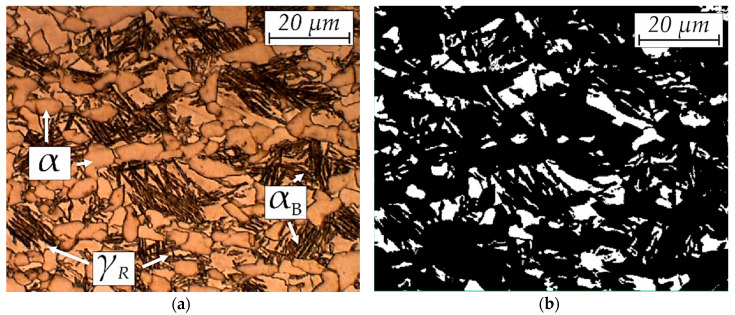
Initial microstructure of the investigated steel: (**a**) optical micrograph and (**b**) binary map of retained austenite (marked as white).

The initial microstructure of investigated steel is presented in Figure 5a. The samples were prepared along the rolling direction. For all the structures listed, both the rolling and stretching directions during the static test were horizontal. The structure was composed of a ferritic-bainitic matrix with retained austenite (Figure 5b). Some balancing of the ferrite and bainite phases was achieved by increasing the carbon content in steel. The steel microstructure was slightly elongated in the rolling direction. Ferrite had a grain thickness of less than 9 μm, which is a consequence of the use of microadditions and thermomechanical treatment resulting in the delayed recrystallization of the austenite during hot rolling [5].

**Figure 6:**
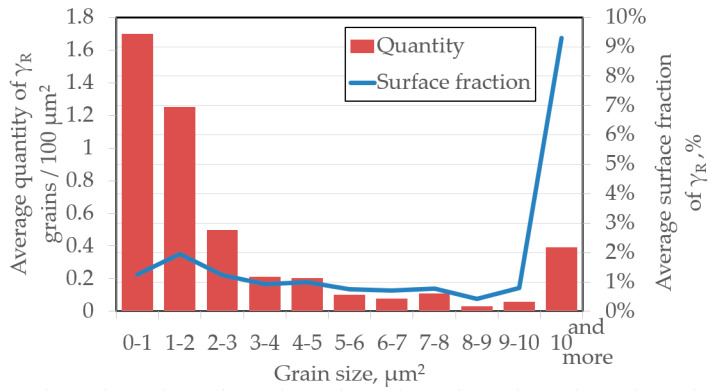
Statistical evaluation of average grains quantity per 100 μm^2^ and the average surface fraction of different size for γ_R_ grains.

Two morphologies dominate in the austenite grains (Figure 5b). Blocky grains were located near the ferrite grains whereas thin layers were located among the bainitic laths. The microstructure was dominated by austenite grains smaller than 3 μm (Figure 6). However, due to their small size, their total surface fraction in the microstructure was ca. 4% whereas the fraction of grains larger than 10 μm was over 9%. Grains with 3–10 μm in diameter were few, amounting to less than 4% of the measured area. The grains larger than 10 μm were just over 2% of the surface fraction.

**Figure 7:**
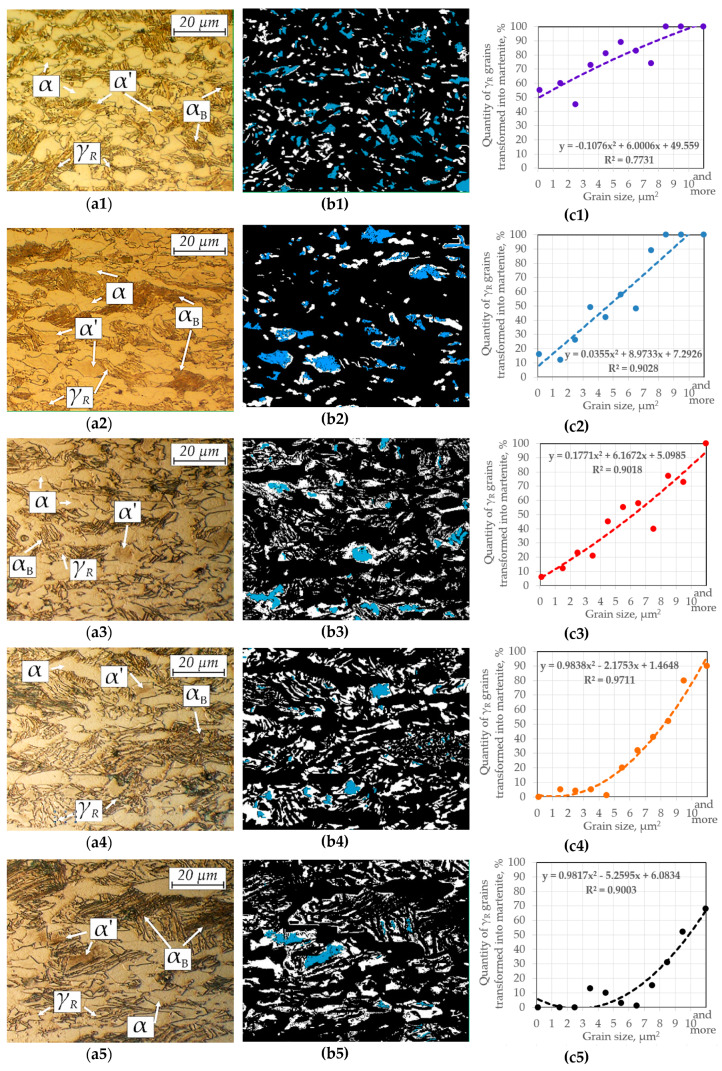
Results: (**a**) optical micrograph; (**b**) binary map of retained austenite (white) and martensite (blue) and (**c**) the quantity of γ that remained stable after deformation; for steel deformed at different temperatures: (**1**) −20 °C; (**2**) 20 °C; (**3**) 60 °C; (**4**) 100 °C and (**5**) 140 °C.

During deformation of the samples, the retained austenite underwent a partial martensitic transformation (Table 3). The fraction of γ_R_ remained after deformation increased with increasing temperature, being 8.6% at the lowest temperature and ca. 19% at the highest. This value was very close to the initial value (19.1%), which means that at 140 °C only a few grains had transformed. The results of the fraction of retained austenite obtained during X-ray analysis in all samples were about 1%–3% of γ_R_ lower than those ones obtained during the image analysis. However, they were in good agreement and the same tendency to increase the fraction of this phase with rising deformation temperature was visible. Figure 7 shows the microstructures of the samples deformed at different temperatures (a), with their binary maps (b) and their statistical analysis for different grain sizes of retained austenite (c). In the microstructure images, the emergence of a new phase—martensite—was clear. Martensite was visible as a darker phase than both ferrite and bainite. Guided by this shade difference, a blue layer was applied to the binary map of the residual austenite indicating the location of the martensite.

The method of applying binary maps is successfully used when determining the share of individual phases in complex microstructures [39,41]. It is clear that the blocky grains were more susceptible to martensitic transformation and thus they exhibited the lower stability compared to thin layers. Interestingly, despite many homogeneous martensite grains, martensitic-austenitic grains dominated. In these structures, retained austenite was located at the grain boundaries, whereas the martensite was present in their center parts. This effect was more pronounced with increasing deformation temperature. At low temperatures, many grains were almost fully transformed (Figure 7a1,b1), whereas at the highest temperatures the strain-induced martensitic transformation produced mainly martensitic-austenitic constituents (Figure 7a5).

As it was indicated earlier, the fact that the proportion of martensite was decreasing along with the temperature increase was very visible. This demonstrated the increase in austenite stability. What is more, the charts (Figure 7) showing the surface fraction of austenite remaining after deformation as a function of grain size testified to a simultaneous increase in the retained austenite stability with a decrease in its grain size. For example, at the lowest temperature 100% of grains above 8 μm^2^ were transformed (it means that the transformation was initiated), while grains of 4 μm^2^ and smaller were only transformed at ca. 50%.

At the highest temperature, the grains above 8 μm^2^ transformed only at 60%, whereas the grains smaller than 7 μm^2^ transformed only sporadically. The results were approximated using a 2nd degree polynomial with the good accuracy expressed as a correlation coefficient (Figure 7c). One could observe a change in the slope of the curve proving the stability of austenite as a function of grain size. Moreover, one could see the shape changes of these curves as the temperature increased. At −20 °C the curve was slightly concave, at 20 °C it almost reached linearity, whereas with increasing temperature to 60–140 °C it showed an increasing convexity. This may indicate that at the elevated temperatures, the stability of retained austenite increased parabolically with increasing the grain size.

### 3.3. Mechanical Stability of Retained Austenite

**Figure 8:**
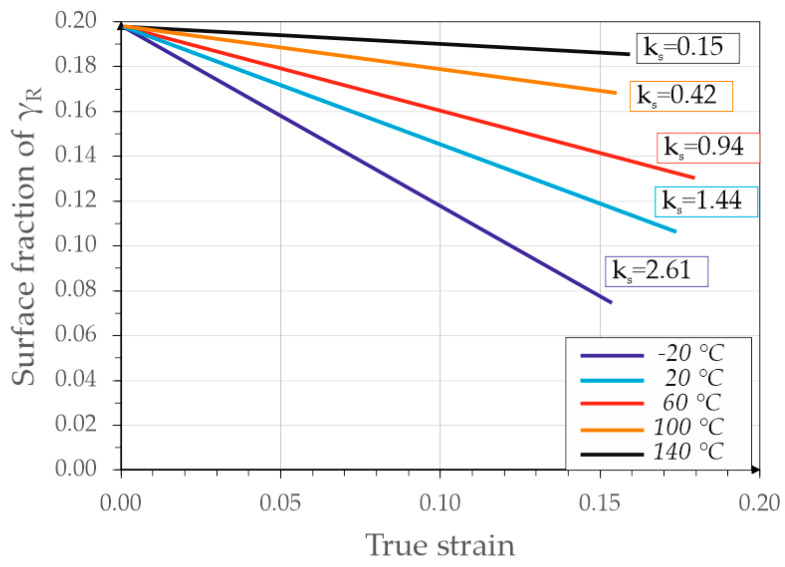
Logarithmic change of the surface fraction of retained austenite as a function of the true strain (at different deformation temperatures), showing the mechanical stability of this phase, expressed by the k_s_ coefficient.

The next step of the analysis was the computational determination of retained austenite mechanical stability. This was performed using both the results of the static tensile test and the microstructure analysis. The austenite stability in the form of the k_s_ coefficient is shown in Figure 8.

**Figure 9:**
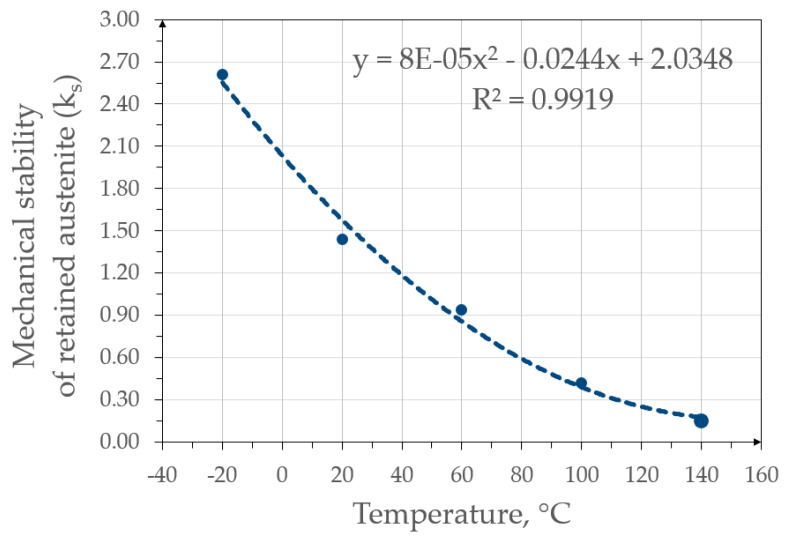
Graph showing the k_s_ coefficient as a function of the deformation temperature.

The slope of the surface fraction–true strain line with respect to the deformation axis determines the mechanical stability of the retained austenite. Along with its reduction, the value of k_s_ decreased, which was associated with an increase in stability. Analyzing the graph, it could be concluded that the mechanical stability of austenite was the lowest at −20 °C and increased with rising temperature until reaching k_s_ equal to 0.15 at 140 °C. The k_s_ values as a function of temperature are summarized in Figure 9. This allows for a very good visualization of the decrease in this coefficient with increasing temperature. Approximation based on the 2nd degree polynomial was imposed on the k_s_ results. It can be seen that the k_s_ changed non-linearly with increasing temperature. The same tendency was observed by Krizan el al. [42]. This indicates that a temperature change in the range of −20–20 °C caused a greater increase in the γ_R_ stability than the subsequent increases in temperatures by 40 °C intervals.

**Figure 10:**
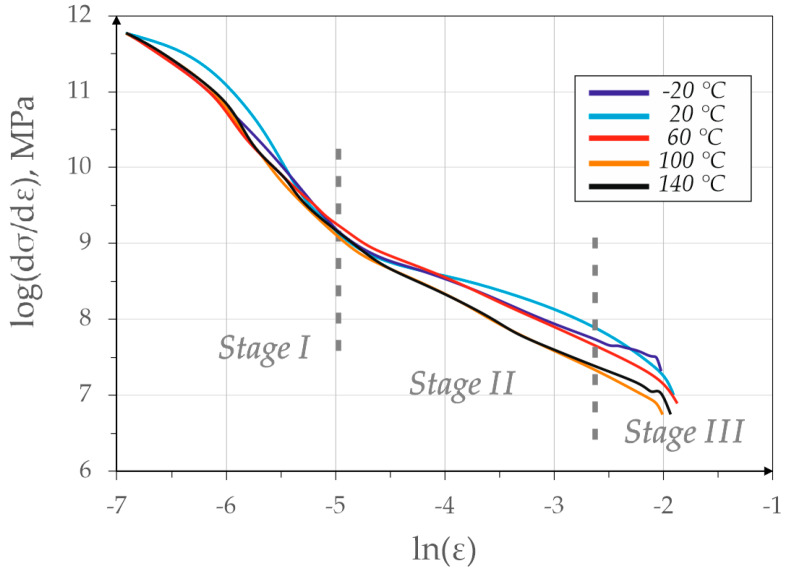
Jaoul–Crussard analysis of the strengthening stages for the samples deformed at different temperatures.

Figure 10 shows the graphs related to the Jaoul–Crussard analysis [34,35]. The successive strengthening phases can be classified as follows:

- Phase I—homogeneous deformation of ferrite matrix by mobile dislocation slip near austenitic-bainitic regions; the hardening is characterized by a high rate at this stage,
- Phase II—limitation of the fall of work hardening rate with little deformation of ferrite and most intense strain-induced transformation of retained austenite into martensite (mainly the block grains);
- Phase III—deformation of ferrite, bainite and strain-induced martensitic transformation of the smaller retained austenite grains; the deformation of structural constituents is accompanied by dynamic recovery [35].

The 3-step strengthening is best seen at deformation temperatures of 20 and 60 °C (Figure 10). In all cases, a similar slope of the curve in the 1st hardening stage is visible—most likely corresponding to the strengthening of the ferritic matrix through a dislocation slip and deformation of the bainitic ferrite. In the case of samples deformed at −20–60 °C the second stage of hardening can be distinguished resulting in lower internal stresses [43]. This stage is attributed to the occurrence of strain-induced martensitic transformation. In the case of samples deformed at 100 and 140 °C, there was no clear II hardening stage due to the poor occurrence of martensitic transformation.

The phase III of the strengthening consisting mainly of the deformation of ferrite, bainite and martensite with accompanying dislocation slip can be distinguished in the sample deformed at 20 °C. This stage has a clear dependence conditioned by a large amount of martensite in the steel structure. For the curve obtained at −20 °C, the lack of the III strengthening stage was found, which was due to the premature rupture of the strongly strengthened sample at smaller strains [35].

## 4. Discussion

So far many studies describe the effect of deformation on the martensitic transformation of the γ_R_ phase at room temperature addressing the corresponding microstructure–property relationships [44,45,46,47]. The impact of deformation temperature on the mechanical stability of retained austenite has so far been barely described in the literature for TRIP steels [31,33]. Studies on the mechanical stability of this phase under different temperature conditions have also been described for medium-Mn steels [48,49], high-Mn steels [50] and quenching and partitioning steels [51].

In the case of TRIP steel, up to now tests of γ_R_ stability at various deformation temperatures were performed for the following ranges: −20–40 °C [21], 20–300 °C [31], 15–110 °C and [32] 25–100 °C [33]. However, in none of the cases did the carbon content of steel exceed 0.2 wt %. One can know this element has a huge impact on the stability of retained austenite. It is visible that until now reported research has usually focused in a selected temperature range. It is important to test and analyze changes in the mechanical stability of this phase over the entire temperature range to which the material is exposed. For this reason, in this work, the deformation temperature range includes negative (−20 °C), room (20 °C) and elevated (60–140 °C) temperatures.

During the research, similar dependencies as in previous works were revealed [21,31,32,33]. The austenite stability increased intensively with increasing temperature, which was confirmed both on the basis of image analysis and X-ray analysis. The results for both methods showed relatively good agreement although there were limitations of the image analysis [6]. The increase in the share of retained austenite along with the increase in deformation temperature is directly related to the increase in its stacking fault energy (SFE) [52,53]. It is clear that after deformation at the lowest temperature (−20 °C) ca. 45% of the retained austenite remained stable near the neck. At this temperature, the austenite showed the lowest stability. Such a large fraction of this phase remaining after the deformation indicates too high stability. This is caused by the higher carbon content in the investigated steel and results in the reduction of potential mechanical properties. The optimum in this case is the reduction of retained austenite stability. To do this while maintaining the current carbon content, it is necessary to change the isothermal holding time and temperature. This could lead to the formation of a larger fraction of bainite, which will absorb more carbon [17,54].

The results showed an increase in elongation with a corresponding decrease in strength as the deformation temperature increased. This is due to the work hardening exponent changes. At temperatures below zero, a continuous increase in the *n* value is observed, which causes the rapid transformation of the retained austenite and premature rupture of the sample. At room temperature, the rise of *n* is dynamic and reaches its maximum in the first phase of deformation. Then it begins to fall leading to the formation of the neck. However, above 60 °C initially the value of *n* is small to reach a maximum value in the final phase of deformation. This causes further strain localization suppressing by delaying the neck formation. This affects the rise in elongation at the expense of strength during deformation at high temperatures. The observed difference in work hardening behavior can be explained by comparing the stability (i.e., transformation rate) of the retained austenite. The slower rate of transformation at the higher temperatures allows for the instantaneous work hardening exponent value to be sustained at higher strains when compared to the lower temperatures. At the higher strains, samples deformed at the higher temperatures still have some austenite available for transformation, whereas the sample deformed at −20 exhausted its TRIP effect [19,55].

**Figure 11:**
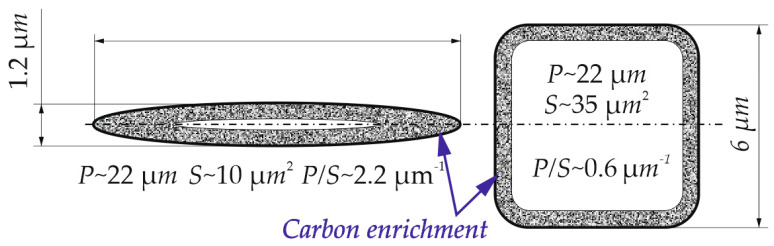
A simplified diagram showing the geometric differences in layer and blocky grain morphologies; S—surface area of grain; P—grain perimeter; ~—approximate value.

The stability of retained austenite depends substantially on its chemical composition (especially carbon content), grain size and morphology [11,12]. Thin layers of γ_R_ localized between bainitic ferrite are more stable than blocky-type grains due to a higher carbon content [49,56]. The grains with thin layers show the higher stability than blocky grains for several reasons. Figure 11 assumes homogenous carbon diffusion perpendicular to the grain border. The form of grain has been simplified into flat figures. For both figures, the area enriched with carbon reaching 0.5 μm deep into the grain was marked. The dimensions of both grains have been chosen so that they have the same circumference. It is evident that due to the large ratio of the circumference to the surface area, the thin-shaped figure has been enriched with carbon on almost the entire surface, whereas the blocky figure has only been enriched to a small extent. The blocky grains have a much larger ratio of volume to the length of the grain boundary. In the case of grains with an extended morphology like thin layers, much less grain volume falls on a given width of the diffusion path and carbon from adjacent alpha phases. This directly influences much faster and more efficient carbon enrichment of these grains.

It should be remembered that the retained austenite with layer morphology usually has a grain size several times smaller than the blocky grains [23,34]. Moreover, these structures are rich in carbon to facilitate the enrichment process. Bainite also creates compressive stresses on retained austenite [57]. All these aspects increase the stability of this phase [58]. During deformation, mainly large grains with the blocky type morphology were transformed. Moreover, only their central part underwent transformation because the long diffusion path prevented the sufficient enrichment in carbon.

However, the change in stability of different retained austenite grain sizes was not proportional during increasing the deformation temperature, which was visible in Figure 7. Comparing the obtained results with the k_s_ values available in the literature, it can be stated that the tested steel at room temperature shows higher stability expressed by k_s_ = 1.4 than steel of the 0.4C-1.5Si-1.5Mn type, for which k_s_ was 2.2–3 depending on the steel matrix [59]. Sugimoto et al. [59,60] produced steels with the value of k_s_ ~ 1.5 for the austenite formed from the tempered martensite matrix. It was visible that with rising temperature, smaller grains show an increase in the stability compared to the larger grains. This relationship has not been properly described in the literature. However, this probably involves the previously described relationship between the surface area and grain volume. The layer-type morphology predominates among smaller grains, whereas the large grains typically have a blocky type shape.

The Jaoul–Crussard analysis revealed that the first stage of strengthening occurred at all test temperatures. It is associated with deformation of ferrite grains and is the strongest at 20 °C. The second stage was especially visible up to 60 °C. Above, it disappeared substantially due to the very weak TRIP effect associated with the high stability of retained austenite. Additionally, stage 3, related to deformation of ferrite, bainite and martensite was leveled when the temperature exceeded 60 °C. This is due to the fact that the amount of martensite had changed at high temperatures.

## 5. Conclusions

The work concerned a detailed study of the microstructural evolution and stability of retained austenite in medium-C TRIP-assisted 0.43C-1.45Mn-0.98Si-1Al-0.033Nb-0.01Ti steel deformed in a temperature range of −20–140 °C. Mechanical properties and stability of retained austenite were determined using the results of static tensile tests and computational approach. The calculations of the k_s_ coefficient and Jaoul–Crussard analysis were performed. The main findings of the present study are as follows:

- Samples deformed at 20 and 60 °C showed the best combination of strength (UTS ~ 890 and 818 MPa, respectively) and plastic properties (TEl ~ 18.3% and 19.1%, respectively) at maximum work hardening exponents of 0.26 and 0.23.
- The deformation temperature and grain size had a great impact on the austenite stability. Its mechanical stability increased with increasing temperature from k_s_ of 2.6 at −20 °C to 0.15 at 140 °C and with the reducing size of the retained austenite grains.
- Blocky grains of retained austenite were more susceptible to the strain-induced martensitic transformation compared to layer-type grains. The martensitic transformation occurred primarily in the central areas of austenite grains resulting in forming numerous austenitic-martensitic constituents. This is due to the lower enrichment of central areas in carbon.
- The increased carbon concentration in the investigated steel resulted in some overstabilization of the retained austenite. As a result, after deformation even at negative and room temperatures, large fractions of this phase remained in the deformed microstructures.
- Plastic deformation and the formation of strain-induced martensite caused the austenite fragmentation. This led to an increase in the number of small retained austenite grains making the microstructure more fine-grained and mechanically stable.
- Jaoul–Crussard analysis showed a decrease in the contribution of the strain-induced martensitic transformation at temperatures above 60 °C to the entire strengthening. There was a lack of clear second phase of the strengthening at elevated temperatures.
- Knowledge on retained austenite stability at various temperatures allows optimization of the chemical composition and heat treatment parameters in terms of the operating conditions of finished components. The increasing stability can also be used to obtain ready-made elements with increased content of this phase. This allows the energy absorption of this material to increase resulting in improved vehicle passenger safety.

## Figures and Tables

**Table 1:** Chemical composition of the investigated steel.

Element	C	Mn	Si	Al	Nb	Ti	P	S	N	O
wt %	0.43	1.45	0.98	1.00	0.033	0.01	0.01	0.004	0.0028	0.0009

**Table 2:** Mechanical properties of steel deformed at various temperatures.

Deformation Temperature, °C	YS_0.2_, MPa	UTS, MPa	TEl, %
−20	536	853	15.8
20	540	889	18.3
60	502	818	19.1
100	490	719	17.1
140	475	702	17.3

**Table 3:** Amount of γ_R_ at various deformation temperatures.

Amount of Retained Austenite at Initial Microstructure *=* 19.1% ^1^				
Deformation Temperature, °C	Sγ_R_, % ^1^	Vγ_R_, % ^2^	Percentage of γ_R_ that Remained Stable Compared to the Initial Microstructure, %	The Smallest γ_R_ Grain Transformed into Martensite during Deformation, μm ^2^
**−20**	8.6 ± 2.1	6	45	<1
**20**	10.5 ± 1.8	8	55	<1
**60**	13.9 ± 1.9	12	73	<1
**100**	15.7 ± 1.5	14	82	1–2
**140**	19.0 ± 1.3	17	99	3–4

^1^ The surface fraction based on image analysis. ^2^ The volume fraction based on X-ray analysis.
